# Integrated Purification Systems for the Removal of Disinfectants from Wastewater

**DOI:** 10.3390/membranes15020043

**Published:** 2025-02-02

**Authors:** Aleksandra Klimonda, Izabela Kowalska

**Affiliations:** Faculty of Environmental Engineering, Wroclaw University of Science and Technology, Wybrzeże S. Wyspiańskiego 27, 50-370 Wrocław, Poland

**Keywords:** pressure-driven membrane process, ion exchange, adsorption, quaternary ammonium compound, benzalkonium chloride, cationic surfactant

## Abstract

The efficiency of integrated treatment systems for wastewater generated during the washing of disinfectant production lines was investigated. The high organic load (COD 2000 mg/L, TOC 850 mg/L) and 300 mg/L of toxic benzalkonium chloride (BAC) make wastewater an environmental hazard that requires advanced treatment. Initial tests on model BAC solutions (in concentrations corresponding to those found in wastewater), using nanofiltration and ultrafiltration membranes, resulted in up to 70% retention of BAC. To enhance purification, ion exchange and adsorption were introduced as post-membrane treatment steps. In the second part of the investigation, membrane modules characterized by the best separation properties were integrated together with macroporous cation-exchange resin and activated carbon into the purification system to treat wastewater. The research carried out showed that the purification of multicomponent wastewater is a complex task. Significantly lower BAC removal (30%) was achieved in membrane processes compared to the model solutions treatment. In integrated systems, the BAC concentration was reduced to 100 mg/L, TOC to 200 mg/L, and COD to 120 mg/L.

## 1. Introduction

Over the past decade, disinfectants have attracted increasing international attention in public health as emerging microcontaminants [1,2,3]. Disinfectant products are most commonly based on alcohols or cationic surfactants (CSs). The latter are gaining increasing popularity because of their non-flammability and lower skin-irritating properties [4].

Surfactants are amphiphilic compounds composed of a hydrophobic tail, which is insoluble in water, and a hydrophilic head containing water-soluble groups [5]. They can be classified as anionic (negatively charged), non-ionic (uncharged), cationic (positively charged), or amphoteric (charge depends on the pH) depending on the hydrophilic part charge, resulting, according to the class, in different properties and applications [2]. A characteristic feature of surfactants is their presence in solutions either as monomers or in aggregated forms, depending on the concentration. The concentration at which molecules begin to aggregate (which is precisely a narrow concentration range) is known as the critical micelle concentration (CMC).

Due to the positive charge of the ionic group in cationic surfactants, these compounds, particularly quaternary ammonium compounds (QACs), demonstrate an ability to adsorb onto negatively charged surfaces such as cell membranes. As a result, they exhibit a biocidal action versus bacteria, fungi, and viruses [6,7]. This phenomenon leads to the biocidal and antistatic properties of CSs, making them applicable in disinfectants and fabric softeners, antistatic agents, herbicides, adhesion promoters in asphalt corrosion inhibitors, dispersants, toiletries, and many others [8,9].

A significant factor driving the dynamic increase in demand for QACs was the COVID-19 pandemic and the associated increase in the need for personal protective products. Due to the high efficacy of QACs in deactivating the SARS-CoV-2 virus (virus deactivation at an exposure time of 15 s and BAC concentration of 0.2% [10]), surfactants from this group are used as components in agents used for antiviral protection. In 2020, the U.S. Environmental Protection Agency [11] published a list of agents effective in inactivating SARS-CoV-2, comprising 702 products, of which 319 contain quaternary ammonium compounds. The global cationic surfactant market size was USD 0.12 billion in 2023 and is projected to reach USD 0.18 billion in 2032 at a CAGR (compound annual growth rate) of 4.9% [12].

As a consequence of accidental leakages, inadequate wastewater treatment, and specific industrial uses such as antifouling coatings or remediation materials, cationic surfactants have been detected in both freshwater and marine environments [13]. The presence of QACs has been reported in effluents from wastewater treatment plants, sludge, surface waters, sediments, groundwater, and drinking water [2,14]. Although the concentration of surfactants in domestic wastewater is rather low (<20 mg/L) [15], for some industrial wastewater it can reach hundreds of mg/L [16]. For example, Aboulhassan et al. [17] reported surfactant concentrations in effluents from a microelectronic factory exceeding 900 mg/L. In addition to the well-documented toxicity to aquatic organisms, it is important to note that the persistent presence of disinfectants in water bodies can facilitate the development and spread of resistance to disinfecting agents. Furthermore, such conditions can also accelerate the emergence and proliferation of antibiotic resistance [18].

Among the methods used for the removal of surfactants from solutions are coagulation [17], biological methods [19], foaming [20], chemical and electrochemical oxidation [21,22], sorption techniques [23,24], ion exchange [25,26,27], and membrane processes [28,29]. However, for most surfactants, removal by conventional processes is often a multifaceted issue, and the efficiency strongly depends on the type of surfactant, its properties, or the presence of other substances.

For example, the chemical coagulation process using coagulants such as iron or aluminum salts is effective for the removal of anionic [17] or non-ionic surfactants [30]. However, in terms of cationic surfactant removal, conventional coagulants are ineffective due to the similar charges of the coagulant and cationic surfactants. Moving forward, biological treatment application is strongly limited by the surfactant type and concentration. In the activated sludge process, the presence of anionic surfactants (LAS, SDS) at concentrations above 200 mg/L inhibits the metabolic processes of microorganisms [31], while the cationic surfactants, well-known for their ecotoxicity, inhibit activated sludge at concentrations 10–40 mg/L [32]. It should be added that surfactants undergo biodegradation after the depletion of the COD fraction available to microorganisms [33].

Membrane separation processes have been widely investigated in terms of separation of all types of surfactant. Techniques operating at reduced transmembrane pressure allow a high degree, although not complete, of surfactant removal from aqueous solutions. Korzenowski et al. [28] reported that anionic surfactant rejection exceeded 95% and 92% when commercial NF membranes (Filmtec Corp., polyamide thin-film composite) were used for model solutions and wastewater purification, respectively. Nguyen et al. [34] applied UF membranes (MWCO (molecular weight cut-off) 10–30 kDa) to non-ionic surfactant Marlophen NP5 (concentrations in the range 1.5–4.4 CMC, CMC = 27.4 mg/L [35]) and reported a removal from 84 to 100%.

Processes used for the removal of surfactants from water solutions include adsorption and ion exchange. Yakout and Nayl [36] found that CTAB removal (feed solution 364 mg/L) with powdered activated carbon (PAC, 1 g/dm^3^) improved with longer contact time. Adsorption was fastest in the first minutes, achieving 90% removal in 10 min, with equilibrium reached after 40–50 min. In the study [37], the adsorption efficiency of CTAB on granular carbon (1 g of GAC/L and 40 mg of CTAB/L) was also investigated. The study showed that the removal efficiency of CS increased with a longer contact time, reaching 36% of removal after 25 min and a maximum level of removal of approx. 70% after 300 min.

The ionic nature of surfactants allows their removal in the ion-exchange process. The first attempts to use anion-exchange resins to remove organic ions, including surfactants, began in the late 1960s [38]. Yang et al. [39] tested the effectiveness of 11 resins with surface areas from 460 to 880 m^2^/g to remove an anionic surfactant (SDBS). The ion-exchange capacity increased with the surface area of the resin, from 3475 mg SDBS/g (for the resin with an effective surface area of 460 m^2^/g) to 4986 mg SDBS/g (for the resin with an effective surface area of 880 m^2^/g), respectively.

Current research focuses on the application of membrane treatment as the primary technique for treating wastewater containing disinfectants. Due to their highly toxic effects (even at low concentrations), emphasis was placed on maximizing the removal efficiency of cationic surfactants. Despite the numerous advantages and superiority of membrane technologies over conventional processes, their use as unit processes may be insufficient for multi-component wastewater. To enhance permeate quality, additional purification processes involving adsorption and ion exchange were proposed. This study introduces, for the first time, the concept of applying integrated processes (UF + IE, UF + PAC) to the purification of cationic surfactant-contaminated wastewater. Adsorption was chosen because of its effectiveness in removing hydrophobic and organic compounds, and ion exchange was selected for its high efficiency in bonding with ionic pollutants, including cationic surfactants.

## 2. Materials and Methods

### 2.1. Research Plan and Scope

**Figure 1:**
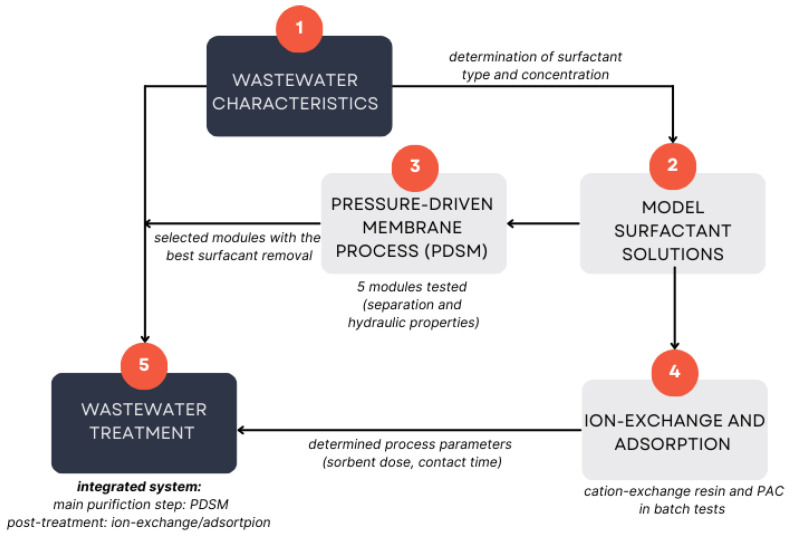
Experimental studies plan.

The research was divided into stages, and the results obtained at each stage allowed the design of an integrated system for wastewater treatment (Figure 1). On the basis of the wastewater characteristics and the type of surfactant, preliminary studies were conducted using model solutions of this surfactant at concentrations similar to those found in the wastewater.

In the first stage, membrane separation tests for surfactants were conducted. Four polymeric modules and one ceramic tubular module were selected for the study, each with pore sizes smaller than the size of the surfactant molecules, which was expected to ensure high separation. Two modules demonstrating the best separation properties were selected for further research.

The second stage of the study focused on the application of ion exchange and adsorption (with powder-activated carbon) to remove surfactants from aqueous solutions. The purpose of the test was to evaluate the suitability of cation-exchange resins and activated carbon for the removal of surfactants from model solutions. On the basis of the obtained data, the process parameters (sorbent/resin dose and contact time) were determined for further studies.

The third and main stage of the investigation focused on the sequential treatment of wastewater from the disinfectant production plant. The primary purification process was membrane filtration. The permeate obtained from the pressure-driven membrane process (PDMP) was further purified by using ion-exchange resin or adsorption.

### 2.2. Wastewater

The wastewater was delivered from the washing stage of the production line of the commercial disinfectant with the use of compressed steam. According to the data available from the producer, the commercial disinfectant is a mixture of 2-phenoxyethanol, N,N-bis-(3-aminopropyl) dodecylamine, and benzalkonium chloride (BAC). Characteristics of industrial wastewater included the following parameters: total organic carbon (TOC, measured using the high-temperature catalytic combustion method, apparatus 550 TOC-TN, Hach, Loveland, CO, USA), turbidity (optical apparatus 2100N IS, Hach, USA), chemical oxygen demand (COD, cuvette tests TNT, Hach, USA, with a concentration range of 0–1500 mg O_2_/L with a DR3900 spectrophotometer, Hach, USA). Potentiometric measurements (785 DMP Titrino, Metrohm, Herisau, Switzerland) were employed for surfactant concentration measurements.

### 2.3. Model Solutions

**Figure 2:**
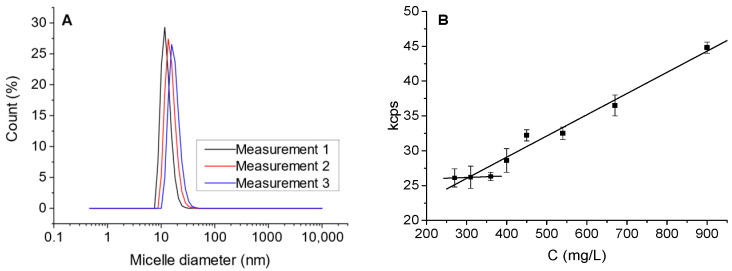
Benzalkonium chloride micelle size distribution (**A**) and the dependence of light scattering on surfactant concentration (**B**) (CMC determined as the intersection point of the approximating lines).

The model solutions of the cationic surfactant were prepared from distilled water and benzalkonium chloride (BAC, 80% purity). Surfactant micelle size distribution and CMC (Figure 2) was determined via the dynamic light scattering method (Zetasizer Nano ZS, Malvern, UK) and amounted to 11.8 nm and 350 mg/L, respectively. Surfactant concentrations of 250 and 500 mg BAC/L were prepared, corresponding to the surfactant content measured in the wastewater. Moreover, it allowed us to obtain both monomeric and micellar solutions (BAC CMC = 350 mg/L). A UV Mini1240 UV-Vis spectrophotometer (Shimadzu, Kyoto, Japan) with the ability to acquire spectra in the range of 190 to 1100 nm was used for BAC concentration measurements.

### 2.4. Membrane Separation

The studies utilized ultrafiltration and nanofiltration polymeric tubular modules of the AFC and ESP series (PCI Filtration Group, Hampshire, UK) and the ultrafiltration ceramic module (CéRAM INSIDE^®^, Tami Industries, Nyons, France), with their characteristics summarized in Table 1.

**Figure 3:**
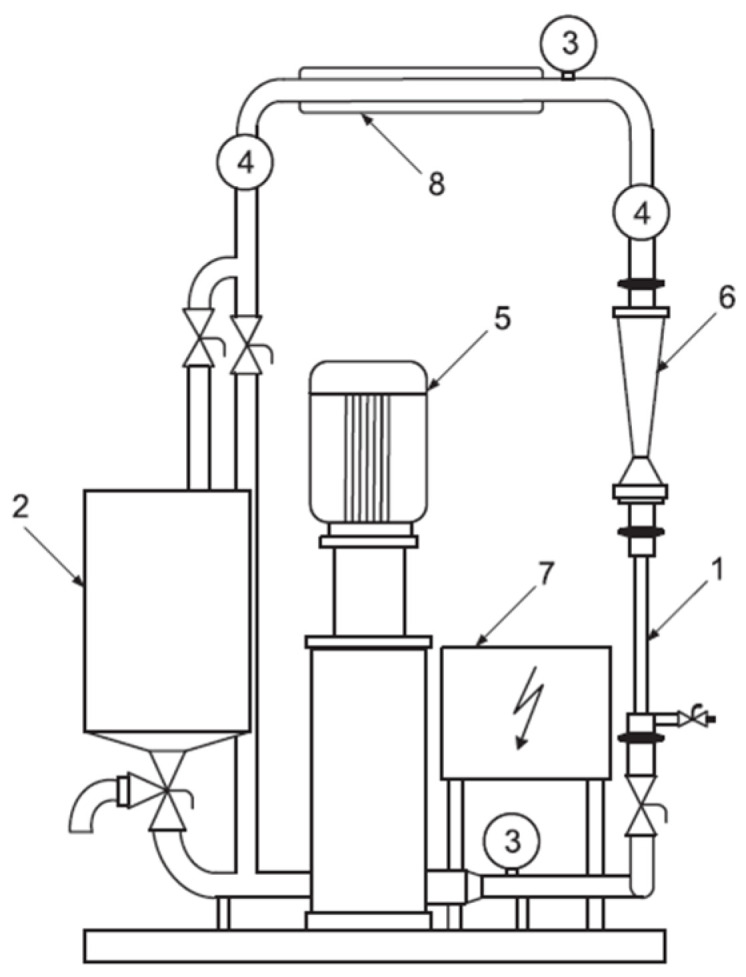
Laboratory set-up: 1—membrane module, 2—feeding tank (10 L), 3—manometer, 4—thermometer, 5—pump, 7—control panel, 8—cooler.

Pressure-driven membrane processes were performed using a semi-pilot installation made of stainless steel (Figure 3). The volume of solutions subjected to purification was 8 L, with transmembrane pressures of 0.3 MPa applied. The studies were carried out under two operating regimes. For the model solutions, recirculation of the retentate and the permeate to the feed tank was employed to maintain a constant concentration of cationic surfactant in the system, and the process was run for 120 min. For the wastewater, the permeate was removed from the system, resulting in a decrease in the volume of the solution within the system over the course of the process. The processes were performed to achieve a two-fold decrease in the feed solution volume.

### 2.5. Ion Exchange and Adsorption

The ion exchange and adsorption processes were carried out in 2 L reactors containing 1 L of the solution and charged with resin doses of 1 or 2 g and PAC doses of 0.5 or 1.0 g. The reactors were placed in the Velp Scientifica FC6S flocculator and stirred at a rotational speed of 150 rpm, ensuring uniform distribution of the resin/sorbent within the entire volume of the solution. Samples were collected for BAC concentration measurement after pre-defined mixing periods. Batch experiments were limited to 2 h to reflect typical industrial treatment cycles, balancing efficiency and cost considerations.

The C150H (Purolite, Gdynia, Poland) was selected for ion exchange. This resin, composed of polystyrene crosslinked with divinylbenzene, has a macroporous structure and a grain size of 600–850 µm. As a conventional adsorbent powder, activated carbon (PAC, Chemviron, Feluy, Belgium) was applied.

## 3. Results

### 3.1. Model Solutions

#### 3.1.1. Pressure-Driven Membrane Processes

**Figure 4:**
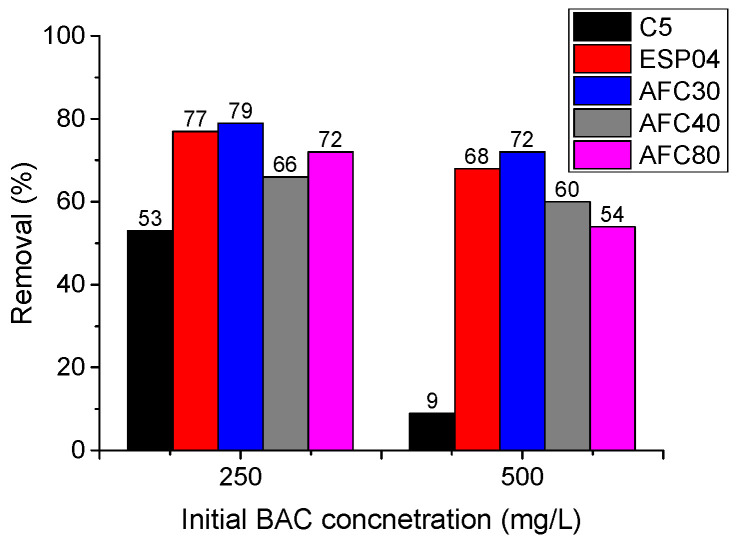
Removal of BAC (model surfactant solutions, filtration time 120 min, TMP = 0.3 MPa, T = 22 °C).

In preliminary studies involving BAC model solutions, modules with the best separation properties were chosen for wastewater treatment. The retention of the surfactant obtained during the 120 min filtration cycles is presented in Figure 4. The hydrophilic ceramic module C5 with the highest MWCO value exhibited the lowest BAC separation (53% for 250 mg/L and 9% for 500 mg/L) among all the modules tested, highlighting the impact of membrane MWCO on surfactant separation in the sieve mechanism. It should be noted that the C5 module was the most hydrophilic of all modules tested, which also played a role in the worse BAC separation. Polymeric modules characterized by a more compact structure and lower hydrophilicity, i.e., AFC80, AFC40, and AFC30 achieved 72, 79, and 66% removal of BAC from solutions 250 mg/L, respectively. When the initial concentration was higher, that is, 500 mg/L, the rejection was lower and amounted to 54, 72, and 60%, respectively. The ESP04 module ensured similar efficacy (77% for 250 mg/L and 68% for 500 mg/L) compared to those observed for the NF modules. The variation in BAC rejection can be attributed to the properties and behavior of BAC in solution. At low surfactant concentrations (below the CMC, that is, 250 mg/L), the separation is driven by the adsorption within the porous structure of the membrane. For solutions with concentrations of 250 and 500 mg/L, micelles and pre-micelles are present; however, as the surfactant concentration increases, the number of free monomers (which remain outside the micelle structure) also increases, and the adsorption capacity of the membrane becomes exhausted, affecting deterioration in separation efficiency. A more pronounced decrease in the removal of BAC shown by the ceramic module C5 with an increase in the initial concentration was due to its larger pores and probably due to the reduced hydrophobic interactions between the membrane material and the BAC molecules.

It should be noted that due to its greater pore size, the hydraulic performance (Table 1) of the ESP04 module was significantly higher than that of the AFC series modules. The distilled water flux of the UF ESP04 module at TMP = 0.3 MPa was three-fold higher than that of the AFC40 and AFC30 modules and nearly ten-fold higher than that of the AFC80 module.

The AFC nanofiltration modules differed in average pore size values (Table 1). Contrary to expectations, the use of the AFC80 nanofiltration module, characterized by the smallest pore size (0.38 ± 0.24 nm) among all NF modules, did not result in the best BAC separation. Retention was 7–18% lower than those observed with the other AFC30 module. Furthermore, the AFC80 module demonstrated a lower separation of BAC compared to the ESP04 ultrafiltration module.

In the studies by Otero-Fernández et al. [41] on the properties of AFC nanofiltration modules produced by PCI Filtration Group, it was shown that the AFC40 and AFC30 modules exhibit a narrower pore size distribution than the AFC80 module. The pores of the AFC80 module are characterized by greater size variability than those of the other AFC modules, which may explain its poorer separation of BAC. The presence of pores in the AFC80 module structure with sizes ranging from 1.0 to 1.6 nm could facilitate the permeation of linear BAC monomers, resulting in incomplete purification.

Ultrafiltration and nanofiltration modules, characterized by the highest BAC separation from model solutions at concentrations of 250 and 500 mg/dm^3^ (70–80% for AFC30 and 68–78% for ESP04), were selected for the sequential purification system.

#### 3.1.2. Ion Exchange and Adsorption Tests

The strongly acidic nature of the acid residue in the BAC surfactant molecule allowed the selection of ion exchange as an effective post-treatment step. The use of ion-exchange resins allows for greater efficiency in the removal of surfactants from aqueous solutions compared to typical adsorbents. Based on previous studies [42], the strongly acidic macroporous cation-exchange resin C150H (Purolite) was selected for ion exchange. Furthermore, the potential of PAC for the removal of BAC from aqueous solutions was evaluated. The sorption capacities, determined using the linear form of the Langmuir adsorption model, were 416.7 mg BAC/g for C150H and 217.4 mg BAC/g for PAC. These findings are consistent with studies on LAS removal [27], which showed that anion-exchange resins exhibit much higher capacities (0.61–1.21 g LAS/g) than activated carbons (0.027–0.053 g LAS/g).

**Figure 5:**
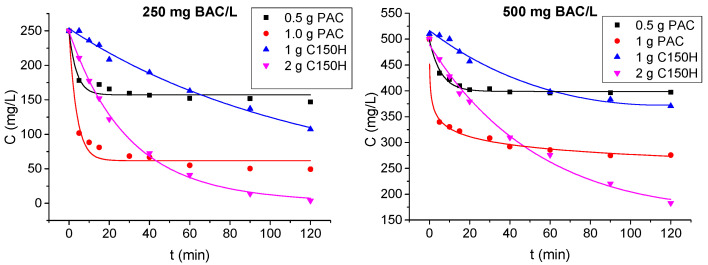
Changes in BAC concentration as a function of contact time with PAC and C150H resin.

Two-hour tests were conducted to assess the effect of contact time and sorbent dose on BAC removal from model solutions with concentrations similar to those in wastewater. Activated carbon was tested at doses of 0.5 and 1 g/L, while resin was tested at doses of 1 and 2 g/L. The adsorption isoplanes (Figure 5) achieved a pseudo-steady state in 30–40 min, which corresponds to the literature data. Kaleta [43] conducted sorption tests of anionic surfactants using activated carbon and reported that complete sorption equilibrium was achieved within 1 h of contact, indicating, however, that a mixing time of 30 min was sufficient to effectively utilize the sorption capacity. In the current study, for the initial concentration BAC of 250 mg/L, its concentration decreased to 150 mg/L and 50 mg/L for PAC doses of 0.5 and 1 g/L, respectively. On the contrary, ion exchange with 2 g/L resin did not reach equilibrium but reduced the BAC concentration to 3.5 mg/L after 120 min, achieving a removal efficiency of 98.6%. For the feed solutions of 500 mg of BAC/L, the concentration decreased to 400 mg/L and 300 mg/L for PAC doses of 0.5 and 1 g/L, respectively, corresponding to removal efficiencies of 20% and 40%. However, for the resin dose of 2 g/L, the BAC concentration reached 180 mg/L after 2 h, achieving removal of 65%.

### 3.2. Wastewater

The characteristics of industrial wastewater are presented in Table 2. Since the wastewater contained surfactant and other organic components used for the preparation of the product formula, the efficiency of wastewater treatment was analyzed based on three pollution indicators: total organic carbon (TOC), benzalkonium chloride (BAC), and chemical oxygen demand (COD).

**Figure 6:**
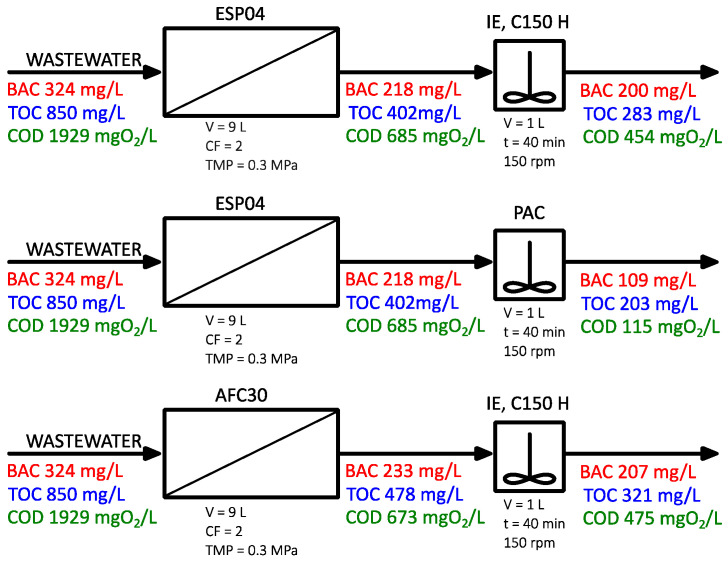
Conceptual diagrams of the integrated treatment systems.

**Figure 7:**
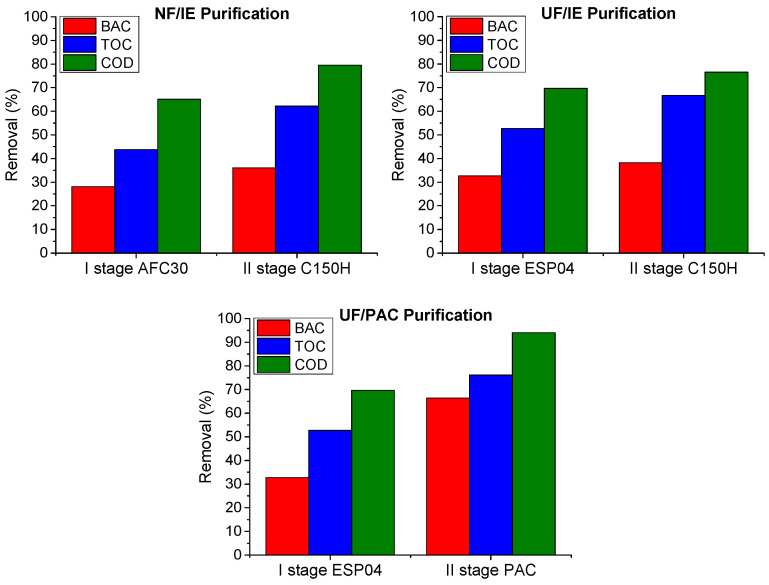
Effectiveness of pollutants removal in integrated purification systems.

The first stage of the treatment involved a membrane process using either a polymeric ultrafiltration (UF) module (ESP04) or a nanofiltration (NF) module (AFC30). Subsequently, the permeate obtained was directed to reactors for post-purification processes, including ion exchange (with C150 H resin at a dose of 2 g/L) and adsorption (using powdered activated carbon, PAC, at a dose of 1 g/L). The wastewater treatment schemes, along with a summary of the parameter values at each treatment stage, are presented in Figure 6, while the percentage removals are illustrated in Figure 7. The UF module yielded wastewater treatment that was several percentage points better than those achieved with the NF module. The most significant change was observed in COD values: the reduction in the concentration of organic compounds was 65% and 70% for the NF and UF processes, respectively. It should be highlighted that a significant decrease in BAC removal efficiency was observed during wastewater treatment (approx. 30%) compared to the results obtained in the model solution tests (approx. 70%). Considering that the changes in the COD and TOC indicators were more pronounced, it can be inferred that other organic compounds present in the wastewater were separated in the PDMP process, potentially influencing the separation of BAC.

**Figure 8:**
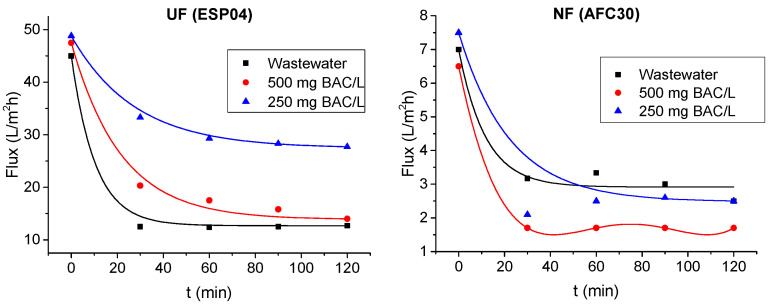
Permeate volumetric flux of ESP04 and AFC30 modules (process time 120 min, TMP = 0.3 MPa).

Membrane filtration of model BAC solutions and wastewater was associated with a decrease in the hydraulic performance of the modules (Figure 8). For both modules tested, the most significant flux decline was observed during the first 60 min of the process, after which the modules achieved relatively stable hydraulic properties. It was observed that from the beginning of filtration, the UF module was the most prone to wastewater fouling (where membrane blocking was influenced by the complex mixture of compounds present in wastewater). The volumetric flux stabilized at approx. 12.5–13 L/m^2^h after 60 min of wastewater filtration, which represents 25% of the flux achieved by this module during the tests with deionized water. However, for the highly concentrated model solution (500 mg/L), after 120 min of the process, this module exhibited a similar volumetric flux value, that is, 14 L/m^2^h, which refers to 29% of the deionized water flux. For the less concentrated model solution of 250 mg BAC/L, the permeability after 120 min was 28 L/m^2^h (58% of deionized water flux). On the contrary, during the tests with the NF module, characterized by a more compact structure and a smaller pore size, wastewater treatment did not produce the most significant deterioration in module permeability. The module exhibited similar hydraulic performance both for wastewater and model solution with a concentration of 250 mg/L, resulting in pseudo-steady flux values after 60 min of the process, approx. 2.5–3 L/m^2^h, representing 36–38% of deionized water flux. The highest fouling intensity was observed for the model solution with a concentration of 500 mg/L. After 30 min of the process, the flux stabilized at 1.7 L/m^2^h, which represents 26% of the initial flux.

**Figure 9:**
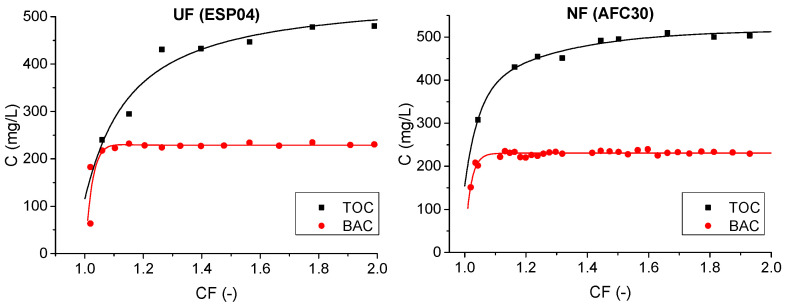
TOC and BAC concentrations in the treated wastewater with the use of ESP04 and AFC30 modules vs. concentration factor (CF, calculated as the ratio of the initial volume of treated wastewater to the final volume of concentrated wastewater; process performed to two-fold concentration of feed solution, TMP = 0.3 MPa).

The changes in BAC and TOC concentrations during the course of the filtration are presented in Figure 9. At the beginning of the processes, the TOC concentration in treated wastewater was approximately 250 mg/L and 300 mg/L for the UF and NF modules, respectively, while the average concentration in the permeate collected during the entire process (after achieving a two-fold concentration of the feed) was 402 and 478 mg/L, respectively. The results obtained indicate that the adsorption of organic compounds in the porous structure of the membrane module played a significant role in the separation mechanism. With the depletion of the modules’ sorption capacity, fouling intensified, leading to reduced separation efficiency as the process depended more on the sieving mechanism. Due to its larger pores and more open structure, the UF module provided superior contaminant separation.

Since wastewater treatment in the membrane process was insufficient (BAC concentration 218 and 233 mg/L, TOC concentration 402 and 478 mg/L for ESP04 and AFC40 module, respectively), the obtained permeates were subjected to post-treatment using ion-exchange resin or PAC. Based on preliminary studies, it was determined that a contact time of 30 min, with doses of 1 g of PAC/L and 2 g C150H/L, may significantly improve the quality of the permeate. After 30 min, the BAC removal efficiency from a model solution was 73% for the PAC and 71% for the C150H resin.

**Figure 10:**
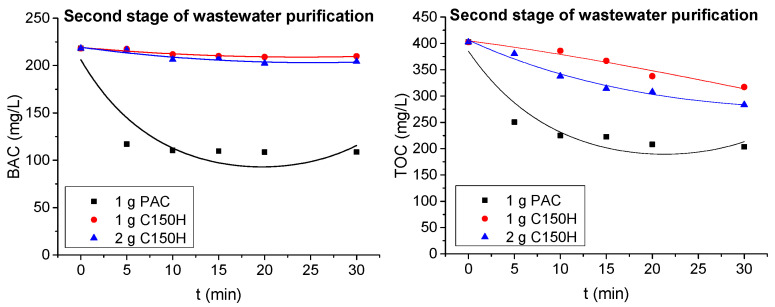
BAC and TOC concentrations during post-treatment with the use of PAC and C150H resin.

Figure 10 presents the changes in TOC and BAC concentrations over the course of the process (UF permeate as a feed solution), for two resin doses and one carbon dose. The application of PAC resulted in better removal efficiency for both types of contaminants studied. It was observed that within just 5 min from the start of the process, activated carbon adsorbed approximately 40–50% of the contaminants, decreasing the TOC concentration from 400 to 250 mg/L and the BAC concentration from 220 to 110 mg/L. With increasing contact time, its effectiveness did not improve significantly. The cation-exchange resin demonstrated minimal effectiveness in removing BAC. Over 30 min of the contact, the surfactant concentration decreased by only 10 mg/L. However, considering the change in the TOC concentration, it was observed that other components (ex. alcohol and other organic compounds) of the wastewater mixture were removed during ion exchange. A decrease in TOC concentration was recorded from 402 to 350 and 320 mg/L for resin doses of 1 g/L and 2 g/L, respectively. It should also be noted that doubling the resin dose (from 1 to 2 g/L) did not result in a significant improvement in the effectiveness of ion exchange.

## 4. Conclusions

- The ultrafiltration (UF) and nanofiltration (NF) modules partially removed cationic surfactant benzalkonium chloride (BAC) from the model solutions. The most effective modules (ESP04 and AFC30) achieved separation efficiencies ranging from 68% to 79%.
- The preliminary adsorption/ion exchange tests on model solutions proved differences in the removal kinetics of BAC. Adsorption on PAC occurred rapidly, reaching equilibrium within minutes, making it suitable for pretreatment or situations requiring short contact times. Ion exchange was less dynamic, requiring extended contact times to reach full capacity; however, the total exchange capacity of the resin was higher than that of activated carbon.
- The efficiency of pollutant removal from wastewater by means of PDMPs was much lower compared to model solutions due to its more complexity. The ultrafiltration module (ESP04) reduced COD, TOC, and BAC by 70, 55, and 30%, respectively. The use of PAC in the post-treatment process improved removal ratios up to 94, 76, and 66%, respectively.
- The integrated purification system proved to be effective in decreasing the concentration of organic compounds in wastewater. However, it should be noted that the surfactant concentration was reduced to a much lesser extent compared to model solutions, indicating that other contaminants present in wastewater are preferentially removed.
- Simple systems based on easy-to-operate membrane processes are preferred. However, for complex wastewater, the two-stage approach remains a practical and effective solution.

## Figures and Tables

**Table 1:** Parameters of membrane modules.

Symbol	Type	Material	Salt Removal, %	Molecular Weight Cut-off, kDa	Pore Size, nm	Deionized Water Flux, L/m^2^h *	Hydrophilicity **
AFC80	RO/NF	PA	80 NaCl	<0.2 [40]	0.38 ± 0.24 [41]	5	hydrophilic
AFC40	NF	PA	40 CaCl_2_	0.2 [40]	0.44 ± 0.07 [41]	15	hydrophilic
AFC30	NF	PA	53 CaCl_2_	0.2 [40]	0.51 ± 0.10 [41]	16	hydrophilic
ESP04	UF	modified PES	-	4	-	48	moderately hydrophilic
C5	UF	TiO_2_	-	5	-	46	highly hydrophilic

* evaluated by the authors, TMP = 3 bar, T = 22 °C, ** According to the manufacturer’s data.

**Table 2:** Characteristics of wastewater.

Parameter	Average Value
pH	10.24 ± 0.95
Conductivity, µS/cm	119 ± 18
Turbidity, NTU	0.8 ± 0.2
BAC, mg/L	324 ± 23
COD, mg O_2_/L	1929 ± 135
TOC, mg C/L	850 ± 27

## Data Availability

All data are presented within the article.
